# Valorization of Brown Seaweed (*Lessonia spicata*): Cellulosic Saccharification for the Development of a Functional Fermented Beverage

**DOI:** 10.3390/foods15010005

**Published:** 2025-12-19

**Authors:** Sebastián Pizarro-Oteíza, Romina Cea, Millaray Aranda, Jéssica López, Pedro Valencia, Erasmo C. Macaya, Fernando Salazar, Oscar Cavieres, Agustín Zavala, Santiago P. Aubourg, Karlo Guerrero, Wladimir Silva-Vera, Paulina Aguirre

**Affiliations:** 1 Escuela de Alimentos, Facultad de Ciencias Agronómicas y de los Alimentos, Pontificia Universidad Católica de Valparaíso, Av. Waddington 716, Valparaíso 2340000, Chile; 2 Laboratorio de Estudios Algales (ALGALAB), Departamento de Oceanografía, Universidad de Concepción, Cabina 3, Casilla 160-C, Concepción 4030000, Chile; 3 Laboratorio de Fermentaciones Industriales (IFELAB), Escuela de Alimentos, Facultad de Ciencias Agronómicas y de los Alimentos, Pontificia Universidad Católica de Valparaíso, Av. Waddington 716, Valparaíso 2360100, Chile; 4 Department of Food Technology, Marine Research Institute (CSIC), c/E. Cabello, 6, 36208 Vigo, Spain; 5 Departamento de Biotecnología, Escuela de Industria Alimentaria y Biotecnología, Universidad Tecnológica Metropolitana, Las Palmeras 3360, Ñuñoa, Santiago 7800003, Chile; 6 Grupo de Investigación en Materiales y Ambiente, Departamento de Química, Universidad Técnica Particular de Loja, Loja 1101608, Ecuador

**Keywords:** Huiro negro (*Lessonia spicata*), cellulosic saccharification, fermented beverage, functional and nutritional properties

## Abstract

This study explored the production of a fermented beverage using Huiro negro (*Lessonia spicata*), a brown seaweed, as a substrate. The cellulosic saccharification (CS) process was optimized via response surface methodology, identifying that the best conditions were 60 U/g of enzyme at 60 °C for 1.9 h, yielding 2.5 g/100 g of reducing sugars. The resulting hydrolysate was fermented with *Lactobacillus* spp. for 48 h at 30 °C and compared with a non-saccharified control. The beverage’s proximate composition, total phenolic content (TPC), flavonoid content (TFC), antioxidant capacity (AC), and *Lactobacillus* spp. viability over 16 days of storage at 4 °C were assessed. CS-treated samples showed a progressive increase in TPC, reaching 126.59 ± 5.58 mg GA/L, which correlated with higher AC. However, no significant differences (*p* > 0.05) were observed in TPC and AC between saccharified and non-saccharified beverages. Notably, the CS-treated beverage achieved significantly higher (*p* < 0.05) *Lactobacillus* spp. counts (10^9^ CFU/mL) compared to the control (10^7^ CFU/mL), maintaining viability throughout storage. While further research is needed to confirm bioavailability and gut health effects, these findings shows that enzymatic saccharification substantially improves fermentation performance and functional properties in *Lessonia spicata*-based beverages.

## 1. Introduction

Functional food production from macroalgae represents a challenge for the food industry, especially in the context of sustainability, diversification of nutritional sources, and cultural food identity [1]. Macroalgae are increasingly recognized as “foods of the future” due to their high nutritional value and unique bioactive compounds, although their full potential depends on effective valorization strategies [2]. Their development must also integrate cultural relevance, as traditional knowledge and culinary heritage promote consumer acceptance [3]. It is essential to bridge regulatory, technological, and social gaps through collaboration between research and public policy, especially now that demographic changes and growing nutritional awareness are driving seaweed consumption, particularly in urban contexts, creating a need to develop attractive products [4,5].

Numerous brown macroalgae are characterized by their high nutritional value. Among them, *Macrocystis pyrifera*, *Lessonia berteroana*, and *Durvillaea incurvata* stand out for their ash, crude protein, and nitrogen-free extract content, respectively. Similarly, *L. spicata* has a high content of total lipids, crude protein, and crude fiber, as well as high concentrations of potassium (K^+^), sodium (Na^+^), and calcium (Ca^2+^) [6]. An understudied option is brown seaweed *Lessonia spicata,* known as Huiro negro, which grows abundantly on the coasts of southeastern Chile [7] due to the topography, strong waves and currents, nutrient availability, water temperature, and shallow intertidal and subtidal zones with low slopes that favor algal growth and the formation of marine forests [8,9].

Various studies have shown the successful incorporation of seaweed derivatives into food matrices such as cookies [10,11], breads [12], extruded snacks [13], and meat products [14], highlighting improvements in fiber, protein, antioxidant capacity, and sensory properties [15]. Although their benefits are clear, innovation in new products is still needed to consolidate their use as functional ingredients. *Lessonia spicata*, rich in polysaccharides, micronutrients, and bioactive compounds, represents a sustainable alternative aligned with the demand for natural foods and responsible management of marine resources [6,16]. This approach favors the circular economy and the valorization of local resources, especially in Valparaíso (Chile), where the use of Huiro negro in functional foods, including fermented beverages, is emerging as a relevant opportunity for innovation and sustainable development [17]. The production of high-quality fermented beverages from *Lessonia spicata* requires an appropriate balance of fermentable compounds and nutrients, with pretreatments playing a key role in this process [18]. Several pretreatment methods such as alkaline treatment, microwave- or ultrasound-assisted extraction, and enzyme-based extraction differ in yield, specificity, time, and cost [19]. Enzyme-based extraction is noted for being more environmentally friendly and providing higher yields due to substrate specificity [19]. Extracted compounds primarily include polysaccharides (alginates, fucoidans, laminarin, carrageenan), proteins, phenolic compounds, pigments, and micronutrients [2]. However, the complex polysaccharides in brown macroalgae are generally inaccessible to most fermentative microorganisms, limiting their direct metabolism [20].

Saccharification is a crucial step in converting polysaccharides into fermentable monosaccharides through acid or enzymatic hydrolysis, enabling the release of sugars such as glucose and other metabolizable compounds [21,22]. Pretreatments with diluted acids or hydrothermal methods enhance sugar release and facilitate fermentation [23]. In the case of *Lessonia spicata*, efficient breakdown of the cell wall is essential to release fermentable sugars and bioactive compounds such as proteins and polysaccharides [24,25]. Enzymes like alginases and cellulases improve polysaccharide hydrolysis, enhance nutrient accessibility and influence fermentation yield and sensory quality [19]. Despite the high cost of biocatalysts, enzymatic saccharification offers greater glucose conversion efficiency [26].

Its performance depends on factors such as enzyme concentration, substrate loading, pH, temperature, and incubation time, which vary among algal species [27]. Since conventional one-factor optimization is limited and time-consuming, response surface methodology (RSM) has become an effective statistical tool to simultaneously evaluate multiple parameters and determine optimal conditions for enzymatic hydrolysis and fermentation of macroalgal feedstocks [28,29]. Furthermore, more studies are needed to analyze the variables of enzymatic saccharification of macroalgae, as there is limited literature on the subject [30,31,32].

On the other hand, starter cultures, including yeasts, acetic acid bacteria, and lactic acid bacteria (LAB), are commonly used in macroalgae fermentation, with temperature, pH, and fermentation time varying according to the selected microorganism [33]. These factors strongly influence the sensory profile and stability of the final beverage [34]. Fermentation with *Lactobacillus* spp. in *Limnospira platensis* and *Alaria esculenta* has been shown to improve color, odor, protein hydrolysis, and sugar metabolization [35,36]. Moreover, fermentation increases volatile compounds such as ketones, alcohols, and furans, enhancing aroma and flavor, although certain aldehydes derived from polyunsaturated fatty acid oxidation can generate undesirable marine odors [37,38]. This process also improves nutritional and functional properties by increasing protein digestibility, vitamin and amino acid synthesis, mineral bioavailability, and bioactive compound formation, while enhancing antioxidant and antimicrobial activities. Refs. [39,40] demonstrated that fermentation of *Alaria esculenta* and *Saccharina latissima* with *Lactiplantibacillus plantarum*, *Saccharomyces cerevisiae*, and Symbiotic Culture Of Bacteria and Yeast (SCOBY) decreased pH and increased lactic acid concentration, generating an acidic environment that improves microbial stability and overall functionality.

According to [41], the use of LAB may enhance the organoleptic characteristics of algae-derived matrices by attenuating intense marine notes and promoting the formation of more pleasant aromatic compounds. Consequently, the lactic fermentation of Huiro negro (*Lessonia spicata*) emerges as a promising strategy for diversifying products and generating differentiated, innovative attributes.

Therefore, the objective of this study is to add value to the brown seaweed Huiro negro (*Lessonia spicata*), which is abundant along the coast of Valparaíso, Chile, by developing a functional and nutritional fermented beverage produced through lactic fermentation and by evaluating the impact of the cellulolytic saccharification (CS) process on its properties.

## 2. Materials and Methods

### 2.1. Raw Materials

**Figure 1:**
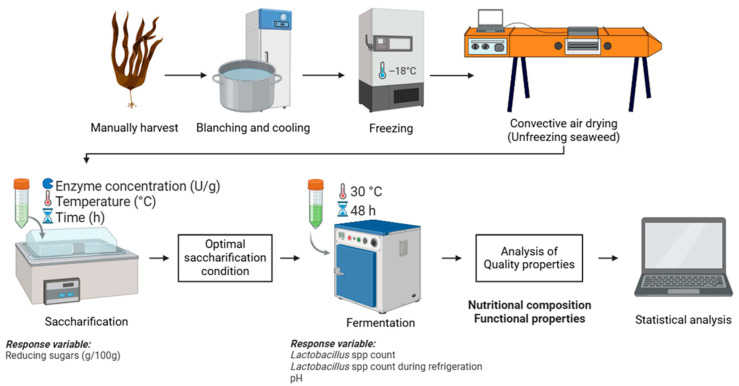
Experimental workflow for the enzymatic saccharification and fermentation of *Lessonia spicata*.

Brown seaweed *Lessonia spicata* was manually harvested in the Valparaíso city coast (33° S, 71° W), Valparaíso Region, Chile. *L. spicata* thallus was stored at 3–5 °C for the following stages. The blanching procedure was carried out according to [18] then the fronds were cut into regular pieces of 3 cm × 0.5 cm (0.2 cm thickness) to facilitate use, transport, and storage. *Lessonia spicata* was finally stored at −18 °C. The methodological scheme is summarized in Figure 1.

### 2.2. Convective Drying Process

The preserved samples were thawed for 24 h at 3.0 ± 0.1 °C prior to drying. A 150 g sample was placed on a rack tray and dried in a convective hot-air tunnel (UOP 8 Tray Dryer, Armfield, Ringwood, UK); see Figure 1. The drying temperature was maintained at 50 °C, with an air velocity of 2.0 ± 0.1 m/s measured using a digital anemometer (air flow), while relative humidity was continuously monitored with a hygrometer. Sample weight was recorded at predetermined time intervals using a digital analytical balance (YMC Co., Ltd., Mount Airy, KY, USA) connected to a computer data acquisition system, which logged the mass variation until a constant weight was reached.

The dried samples of Huiro negro were milled using a stone mill (Fidibus Medium 360, KoMo, Mudgeeraba, Gold Coast, Queensland, Australia), equipped with self-sharpening corundum-ceramic stones (85 mm diameter) and a 360 W motor. The resulting flour was vacuum-packed and stored under refrigeration (3.0 ± 0.1 °C) until use.

### 2.3. Cellulosic Saccharification (CS)

Powdered Huiro negro was employed as the substrate for enzymatic hydrolysis using the cellulase enzyme blend (SAE0020, Sigma-Aldrich, St. Louis, MO, USA, Specific Activity: 1000 U/g) that contained cellulases (EC 3.2.1.4), cellobiohydrolases (EC 3.2.1.91), β-glucosidases (EC 3.2.1.21), and hemicellulases (EC 3.2.1.8) with a density of 1.15 g/mL and an optimum pH of 5.0–5.5. The enzymatic reaction was conducted using 2 g of dried milled Huiro negro substrate suspended in 40 mL of sodium acetate buffer, adjusted to pH 5 in accordance with previously reported protocol [32]. Subsequently, the cellulase enzyme blend was added to initiate reaction under variable conditions, such as enzyme concentration (EC) (10–60 U/g), temperature (T) (40–60 °C), and hydrolysis time (HT) (1.5–3.5 h). Then, reactions were terminated by heating the mixture at 95–97 °C for 10 min, followed by immediate cooling and reducing sugar content quantification.

### 2.4. Reducing Sugar Content (RSC)

Reducing sugar content (RSC) (g/100 g) was quantified as response variable from the cellulolytic saccharification (CS) procedure, as proposed in the literature [32,42]. The 3,5-dinitrosalicylic acid (DNS) method, originally described by [43] and widely used in studies of algal biomass hydrolysis [44,45], was employed to quantify the RSC released during the CS procedure. The RSC was measured at 540 nm using a spectrophotometer.

### 2.5. Fermented Beverage Production

#### 2.5.1. Fermentation Process

The macroalgae hydrolysates were fermented in a defined volume following the methodology previously reported [42]. Commercial lactic acid strains were also used, as described by [36]. Fermentation was carried out at pH 5.0 and 32 °C for 48 h, without agitation. A mixed starter culture (8 mg) of Pro8 Probiotic Strains Yogurt (Fungi Pharm, Santiago, Chile) was used, which included *Lactobacillus bulgaricus* LB-G40, *Streptococcus thermophilus* S1-630, *Lactobacillus rhamnosus* Lr-G14, *Lactobacillus casei* LC-G11, *Lactobacillus paracasei* LPc-G110, *Bifidobacterium bifidum* BB-G90, *Bifidobacterium lactis* BL-G101, and *Bifidobacterium longum* BL-G301. Additionally, a fermented beverage prepared without enzymes (BF W/E) was included as a control.

#### 2.5.2. Monitoring and Quantifying *Lactobacillus* spp. Growth

*Lactobacillus* spp. count (CFU/mL) in the fermented beverage with and without enzyme addition, designated as FB (added enzyme, A/E) and FB (without enzyme, W/E), respectively, was determined starting from an initial inoculum of approximately 10^3^ (CFU/mL). Samples were collected each 8 h at 0, 8, 16, 24, 32, 40, and 48 h to monitor microbial growth across fermentation time. Colony counting was performed following the reported procedure [36]. Man, Rogosa, and Sharpe (MRS) agar (Scharlau, Barcelona, Spain) was used as culture medium of *Lactobacillus* spp. Additionally, pH measurements were taken at the previously mentioned time intervals. Finally, the viable count of *Lactobacillus* spp. was determined in the fermented beverage with enzyme addition, FB (A/E), during storage at 4 °C for up to 16 days.

### 2.6. Quality Parameters

#### 2.6.1. Nutritional Composition

Samples of Huiro negro including fresh, hydrolyzed, and fermented beverages were analyzed to quantify total protein, carbohydrate, lipid, ash, and dietary fiber contents. Moisture and ash contents were determined according to official AOAC methods, following reported procedures [46], which involved drying the samples in an oven at 105 °C for 24 h to measure moisture and incinerating at 550 °C for 12 h to assess ash content. Total carbohydrates were quantified using the phenol-sulfuric acid assay [47]. Crude protein content was measured by the Kjeldahl nitrogen method [48]. Total dietary fiber was analyzed using the enzymatic-gravimetric AOAC method [49]. Lipid content was determined by Soxhlet extraction [50]. All analyses were performed in triplicate and results were expressed on a dry weight basis as means with standard deviations.

#### 2.6.2. Bioactive Compounds

##### Total Phenolic Content (TPC)

To extract phenolic compounds and flavonoids from brown seaweed *Lessonia spicata*, the procedure reported by [51] was carried out. Around 2 g of sample was weighed, and an extract was performed with 20 mL of methanol (60%). The extraction was carried out at room temperature (29 ± 2 °C) for 24 h under dark conditions at 100 rpm in an incubator and shaker. The total phenolic compound content (TPC) was determined as described by [52]. In a microplate, 75 µL of water, 20 µL of 1N Folin–Ciocalteu reagent, and 10 µL of *L. spicata* seaweed extract were added and allowed to stand for 5 min. Subsequently, 30 µL of a saturated Na_2_CO_3_ solution (10%, *w*/*v*) was added and, finally, 120 µL of distilled water. The sample was incubated in the dark at room temperature for 2 h. The absorbance was measured at 760 nm by comparing the results with previously prepared standards using known concentrations of phenolic compounds. TPC results were expressed as mg GAE/L.

##### Total Flavonoid Content (TFC)

The TFC of the extracts obtained was determined following previous methodology [53]. Quercetin was used for the calibration curve. The extracts were diluted (FD:10), and in the microplate 75 µL of methanol was added, followed by 5 µL of 10% *w/v* aluminum chloride and 25 µL of sample, and allowed to stand for 3 min. Then, 5 µL of 1M potassium acetate and 140 µL of distilled water were added. After incubating at room temperature (21 ± 2 °C) for 30 min, the absorbance of the reaction mixture was measured at 415 nm using a spectrophotometer (Multiskan Go, Thermo Scientific, Waltham, MA, USA). The TFC was expressed as mg QE/L. The absorbance of the supernatant was measured in triplicate [54].

#### 2.6.3. Antioxidant Capacity

The antioxidant capacity was measured by DPPH (2,2-Diphenyl-l-picrylhydrazyl radical), ABTS (2,2′-azino-bis (3-ethylbenzothiazoline-6-sulfonic acid)), and FRAP (ferric reducing antioxidant capacity). The DPPH radical scavenging activity of samples was measured according to [55] adapted for microplate assays. A 100 µL of 60 µM methanolic solution of DPPH was added to 100 µL of sample and incubated for 30 min in the dark. The absorbance was measured at 517 nm in a spectrophotometer (Multiskan Go, Thermo Scientific, Waltham, MA, USA) and compared to a Trolox equivalent (TE) calibration curve. The results were expressed as µmoles Trolox equivalents (TE) per liter (µmoles TE/L). The ABTS radical cation (ABTS^+^•) scavenging activity was determined according to [56], with modifications. The ABTS^+^• solution was prepared by mixing equal volumes of 7 mM ABTS and 2.45 mM potassium persulfate solutions and allowing the mixture to react overnight (~16 h) under gentle agitation in the dark at room temperature (21 ± 2 °C). The working solution was adjusted to an absorbance of 0.700 ± 0.02 at 734 nm with methanol. A hundred µL of sample was mixed with 100 µL of ABTS^+^• solution in a microplate well, and the mixture was incubated in darkness at room temperature (21 ± 2 °C) for 7 min. The absorbance was measured at 734 nm using a spectrophotometer (Multiskan Go, Thermo Scientific, Waltham, MA, USA). The results were expressed as µmoles Trolox equivalents (TE) per liter (µmoles TE/L). The FRAP assay was performed according to [57] with some modifications. The ferric tripyridyltriazine (Fe (III)-TPTZ) complex was prepared by mixing 25 mL of sodium acetate buffer (pH 3.6), 2.5 mL of TPTZ solution, and 2.5 mL of FeCl_3_ (20 mmol/L).

A total of 15 µL of sample was mixed with 285 µL of Fe (III)–TPTZ complex, incubated for 30 min in the dark at 37 °C, and the absorbance was measured at 593 nm in a spectrophotometer (Multiskan Go, Thermo Scientific, Waltham, MA, USA) and compared to a Trolox equivalents (TE) calibration curve. The results were expressed as µmoles Trolox equivalents (TE) per liter (µmoles TE/L).

### 2.7. Experimental Design and Statistical Analysis

A 3^3^ experimental design was conducted to evaluate the effect of three independent factors on cellulosic saccharification (CS) processes: enzyme concentration (EC) (10, 35, and 60 U/g), temperature (T) (40, 50, and 60 °C), and hydrolysis time (HT) (1.5, 2.5, and 3.5 h), including three central points used to estimate experimental error and test model curvature (Table 1).

The experimental design followed a Box–Behnken structure with response surface methodology (RSM), which enables the estimation of linear, quadratic, and interaction effects among variables. The order of the experimental runs was fully randomized to minimize systematic bias. Each experimental trial produced a quantitative response variable (Y) corresponding to reducing sugar content (RSC) [58,59]. The data obtained were fitted to a second-order polynomial model (Equation (1)).

(1) $$Y_{i}=b_{0}+\sum_{i=1}^{k}b_{i}X_{i}+\sum_{i=1}^{k}b_{ii}{X_{i}}^{2}+\sum_{i<j}b_{ji}X_{i}X_{j}+\epsilon$$

where Yi was the response (RSC), subscripts i and j ranged from 1 to the number of variables (n = 3), b_0_ was the intercept term, b_i_ values were the linear coefficients, b_ii_ values were the quadratic coefficients, b_ij_ values were the cross-product coefficients, X_i_ and X_j_ were the levels of the independent variables, and ε was the error term. All statistical analyses and graphical outputs were performed using Statgraphics Centurion XVI^®^ software. Model parameters were estimated using the least-squares method, and the overall model significance, individual term effects, and lack-of-fit were evaluated by analysis of variance (ANOVA) at a 95% confidence.

## 3. Results and Discussion

### 3.1. Cellulosic Saccharification

**Figure 2:**
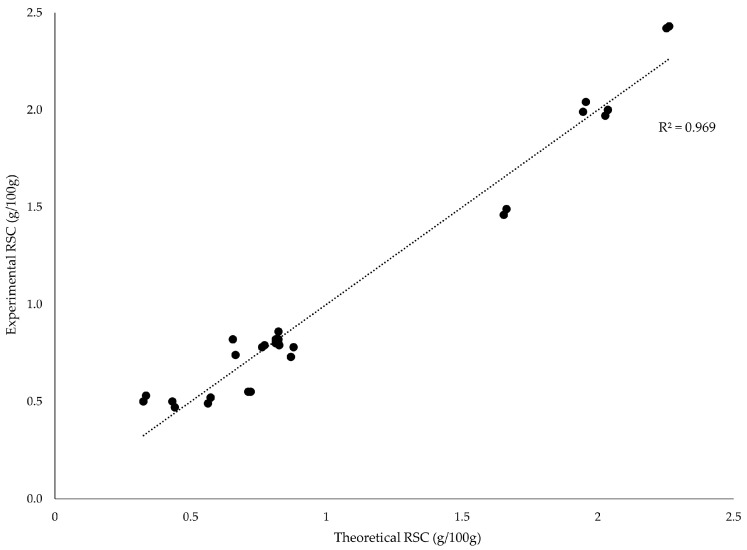
Correlation analysis between experimental and theoretical RSC (g/100 g) values.

**Figure 3:**
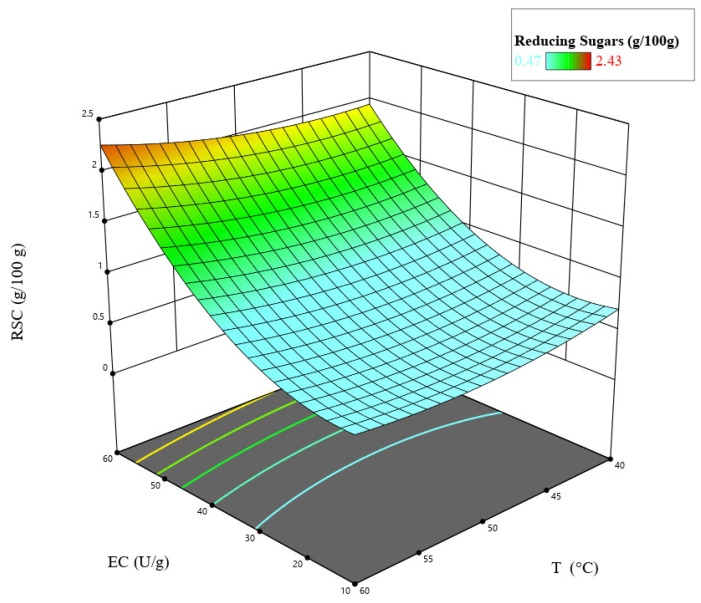
Response surface for RSC (g/100 g) releasing at optimum HT of 1.9 h.

To visualize the combined effects of the independent variables on reducing sugar content (RSC), the response surface model was graphically represented through three-dimensional contour plots and model validation diagrams (See Figure 2 and Figure 3). These graphical tools provide a clearer understanding of the interaction patterns among enzyme concentration (EC), temperature (T), and hydrolysis time (HT), as well as the adequacy of fitted regression.

Analysis of the experimental design using the RSM revealed that the polynomial modeling the hydrolysis phenomenon is a second-order polynomial (Equation (2)):

(2) $$Y_{RSC}=a-b\times EC-c\times T+d\times HT+e\times {EC}^{2}+f\times EC\times T-g\times EC\times HT\;+h\times T^{2}+i\times T\times HT-j\times {HT}^{2}$$

where a = 3.017; b = 3.128 × $\;10^{-2}$; c = 1.258 $\;\times {\;10}^{-1}$; d = 7.736 $\;\times 10^{-1}$; e = 7.108 $\;\times 10^{-4}$; f = 4.550 $\;\times 10^{-4}$; g = 4.800 $\;\times {\;10}^{-3}$; h = 1.105 $\;\times {\;10}^{-3}$; i = 1.375 $\;\times 10^{-3}$; and j = 1.482 $\;\times {\;10}^{-1}$.

In general, Equation (2) showed that EC had a positive statistically significant influence on hydrolysis when increasing from 10 to 60 (U/g) with a 4-fold magnitude (*p* < 0.05), meantime, a similar behavior was shown for T, but with non-statistically significant influence (*p* > 0.05).

Conversely, HT showed a negative influence on RSC releasing when exposure time exceeded 1.5 h of processing, although a negligible impact (*p* > 0.05) was detected. The regression coefficient (β) and *p*-values for each significant factor along with fit statistics are presented in Table 2.

A positive effect of EC was expected due to the direct increase in reaction rates and, therefore, greater hydrolysis [60]. As the enzyme concentration increases, the hydrolysis reaction increases, reflecting a proportional relationship between RSC [35].

On the other hand, the presence of quadratic effects on the mathematical model in Equation (2) would represent the presence of an inflection point. EC and T suggested that there was a maximum hydrolysis point for RSC released, with a positive effect. Nevertheless, above a certain EC, the enzyme activity may stabilize this act being attributable to a substrate saturation. Moreover, above an optimal temperature, the enzyme loses activity, causing a drop in hydrolysis [21].

The quadratic effect of HT indicates that the reaction rate was not constant. Initially, hydrolysis increases, but as the substrate was consumed, the rate decreases [60]. Likewise, a quadratic EC∙T interaction meant that the effect of EC on hydrolysis varied with temperatures, with positive correlation between them. For example, in this study at a low temperature, increasing enzyme concentration may have no significant effect; meanwhile at the optimal temperature, the same increase can produce a dramatic improvement in hydrolysis. For an EC and T interaction, there was a similar trend to the previous interaction, where mutual dependence could be observed. In contrast, at high concentrations, the same level of hydrolysis can be achieved in a shorter time [21,60,61]. The response surface is presented in Figure 3.

**Figure 4:**
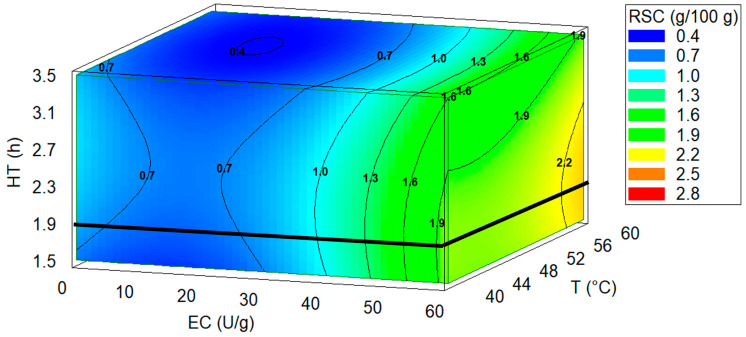
A 3D contour plot showing the combined effect of EC, T, and HT on reducing sugar content (RSC). The continuous line (**─**) represents the optimum HT.

As a primary consequence of experimental analysis, and the best operational conditions to achieve higher concentration of RSC under the levels tested in this study, it is convenient to set the highest EC and T, meanwhile HT must be 1.9 h (See Figure 4). An experimental validation was conducted under the optimal operational conditions predicted by the experimental design, yielding an RSC value of 2.45 g/100 g. This value represents a relative deviation of 6.06% from that estimated by Equation (2), thereby confirming the models’ prediction reliability.

### 3.2. Fermentation Process

**Figure 5:**
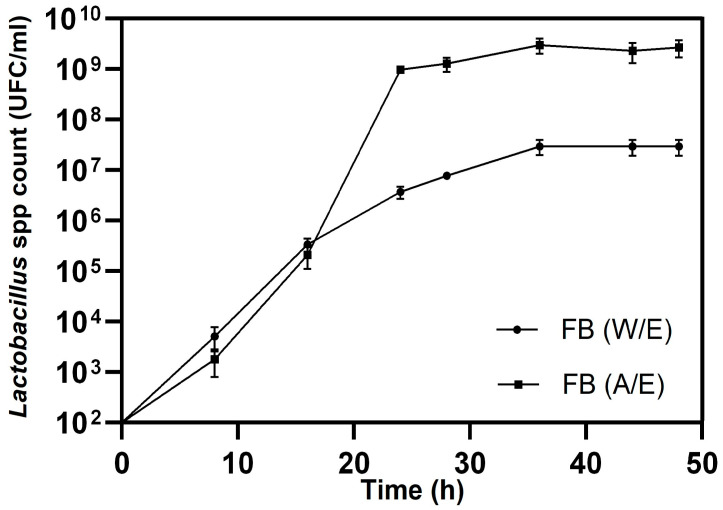
Growth of *Lactobacillus* spp. with and without cellulosic saccharification (FB W/E and FB A/E).

Figure 5 shows the evolution of *Lactobacillus* spp. counts (CFU/mL) in two different treatments: FB (A/E), corresponding to fermentation with enzyme addition, and FB (W/E), corresponding to fermentation without enzymes, during a 48 h incubation period. In both cases, an initial adaptation phase was observed up to 10 h, followed by a growth phase between 10 and 20 h. However, growth differed significantly (*p* < 0.05) between treatments: FB (A/E) increased from 10^5^ to 10^9^ (CFU/mL) and remained stable until the end of the experiments (48 h), while FB (W/E) reached approximately 10^7^ (CFU/mL). In this study, pH ranged from 5.0 to 4.5 in FB (A/E) and from 5.0 to 4.95 in FB (W/E), reflecting the acidification associated with lactic metabolism, where LAB and yeasts convert carbohydrates into lactic acid under anaerobic conditions [62].

These results support the idea that enzyme addition promotes greater biomass production, attributable to greater availability of fermentable sugars derived from the hydrolysis of structural polysaccharides. Furthermore, this agrees with the findings reported by [63], who observed that enzymatic treatment of *Gracilaria corticata* generated the highest RSC and high lactic acid production after 72 h of fermentation with *Lactobacillus acidophilus*. Similarly, [42] showed that enzymatic hydrolysis of red algae improved fermentation by *Lactobacillus casei* and *Saccharomyces boulardii*, highlighting that *L. casei* had a higher growth rate and pH reduction. These findings reinforce the importance of applying enzymatic treatments prior to fermentation to optimize LAB growth and maximize the conversion of complex carbohydrates into biotechnological products of interest. They also coincide with previous studies that highlight the influence of enzymatic pretreatments on microbial kinetics and fermentation yield [64,65]. The incorporation of hydrolysates increases the availability of monosaccharides and, therefore, the biomass production of *Lactobacillus* spp., as also being demonstrated in the fermentation of *Saccharina latissima* and *Ulva rigida*, improving acidification, polysaccharide utilization, and the nutritional enrichment of the final product literature [66,67,68].

In addition, species of the *genus Enterococcus*, widely distributed in aquatic environments, also have biotechnological potential in the production of secondary metabolites, including lactic acid [69,70].

*Lactobacillus* spp. count in BF (A/E) under refrigeration for 16 days remained stable, maintaining a logarithmic load of 10^9^ (CFU/mL), making it stable under storage conditions. This indicates that the integration of enzymatic pretreatment prior to fermentation enhances the growth and metabolic activity of LAB in the biomass of the macroalgae Huiro negro (*Lessonia spicata*), promoting greater biomass accumulation and supporting the sustainable utilization of marine resources for the development of functional foods and high value-added bioproducts.

### 3.3. Physicochemical Properties of Huiro negro

The nutritional properties, bioactive compound, and antioxidant capacity of Huiro negro samples were analyzed in fresh (untreated), dehydrated, hydrolyzed, and fermented beverages (added enzyme and without enzyme).

#### 3.3.1. Nutritional Properties

Before undergoing hydrolysis and fermentation treatments, the Huiro negro samples were characterized in terms of moisture, fat, protein, ash, total dietary fiber, and available carbohydrates with values of 87.5 ± 0.03, 0.19 ± 0.09, 1.68 ± 0.25, 1.81 ± 0.01, 8.71 ± 3.65, and 0.23 ± 0.02, respectively. The fresh samples were air dried and milled, and then resuspended, one part was reserved and used directly as a fermentation beverage (FB W/E), and the other fraction was enzymatically hydrolysate (FB A/E). Table 3 shows significant changes in the composition across treatment.

Fermentation treatments resulted in samples with high moisture levels due to the reconstitution carried out during formulation, which produced lower values for solid components, approaching those of the untreated Huiro negro samples. In this sense, to facilitate comparison among resuspended samples, wet basis was used.

Table 3 shows that fat content is strongly influenced by the treatment applied to Huiro negro. In the hydrolyzed sample, fat levels were lower than hydrolyzed and fermented treatments, maybe due to the accumulation of short-chain fatty acids (SCFA) during fermentation, in this regards, macroalgae fermentation has been shown to increase the production of short-chain fatty acids, such as acetate, propionate, and butyrate. Studies indicate that species such as *Laminaria japonica*, *Pachymeniopsis elliptica*, and *Enteromorpha crinite*, when subjected to anaerobic fermentation, generate concentrations of up to 15.2 g/L of volatile fatty acids after three days of incubation [71]. Similarly, *Ulva rigida* and *Gracilaria fisheri* fermented in vitro with human fecal microbiota showed a significant increase in total SCFA production, reaching between 29.4 and 35.4 µmol/mL compared to 17.9 µmol/mL in the control [72].

These results suggest that fermentation reconfigures the lipid profile of macroalgae towards short-chain metabolites, with potential functional application in intestinal health and biotechnological processes, which could explain the increase in our samples of fermented beverages with and without cellulosic saccharification (CS). Similarly, studies on fermented algae beverages describe changes in the lipid fraction attributable to microbial action and the emulsification of membrane lipids [73].

Table 3 indicates that protein, ash, and total dietary fiber showed no significant differences (*p* > 0.05), irrespective of treatments. Despite decreasing protein value, this behavior was not statistically significant (*p* > 0.05), even though their use was expected because it serves as a nitrogen source. This behavior suggests that nitrogen consumption was low enough to been reflected as statistical differences.

Ash content was expected to have no statistically significant variations among treatments because it servesas a trace element supply, and their consumption during fermentation is marginal and even undetectable. Studies on *Lessonia* spp. have reported similar patterns, indicating that mineral variation is influenced by both the stage of algae development and environmental conditions, season, and location of origin [6].

According to the results, the total dietary fiber values of the hydrolyzed and fermented samples were low (0.81 ± 0.03–0.90 ± 0.02 g/100 g), indicating the degradation of structural polysaccharides such as alginates, fucoidans, and laminarins, which are converted into soluble oligosaccharides. This behavior has been widely reported in studies on the enzymatic hydrolysis of brown algae polysaccharides. Conversely, the total available carbohydrates decreased significantly in both treatments (*p* < 0.05), as expected, since carbohydrates serve as the main carbon source during fermentation, a process typically associated with increases in microbial biomass [74].

#### 3.3.2. Bioactive Compounds and Antioxidant Capacity

The total phenol content of Huiro negro samples showed significant (*p* < 0.05) variations depending on the processing stage (Table 4). The total phenolic content (TPC) was highest in the untreated sample (156.23 ± 3.04 mg GAE/L sample) and decreased significantly after drying (99.46 ± 0.63 mg GAE/L sample). This pattern of partial loss of phenols with heat treatments is consistent with multiple studies showing that heat drying causes degradation or transformation of phenols in macroalgae, with greater loss when high temperatures or prolonged times are used [75].

Enzymatic hydrolysis of carbohydrates, corresponding to cellulolytic saccharification, increased the RSC and generated an intermediate TPC value. This indicates that cellulolytic action released phenolic compounds previously bound to the cell wall and therefore inaccessible to solvent extraction. Interestingly, fermentation with enzymatic pretreatment, also based on cellulosic saccharification (FB A/E), partially recovered the phenolic content (126.59 ± 5.58 mg GAE/L) compared to fermentation without enzyme addition (FB W/E). This suggests that enzymatic activity favored the release of phenols bound to the matrix, such as esters or phenol-polysaccharide complexes, or protected fractions susceptible to oxidation during fermentation. This interpretation is consistent with several studies on microbial and enzymatic biotransformation of phenolic compounds, which indicate that hydrolysis and certain fermentations can promote the release and even an apparent increase in measurable phenols, depending on the microbial strain and the enzyme set used [76]. A similar increase has been reported in fermented algae due to the hydrolytic activity of lactic acid bacteria on polysaccharide matrices [34]. In addition, recent research on macroalgae fermentation describes the partial recovery of phenolic compounds after controlled enzymatic and fermentative processes [74].

The total flavonoid content (TFC) showed a different behavior to that of total phenols (Table 4). The TFC increased progressively during processing, fresh samples presented the lowest level (37.82 ± 0.18 mg QE/L), while drying increased the TFC to 61.52 ± 1.00 mg QE/L and hydrolysis increased it even further (111.41 ± 0.73 mg QE/L); fermented beverages maintained high values (112.10–113.53 mg QE/L), no significant differences were observed between the two conditions (*p* > 0.05). This is because the greatest release of flavonoids occurred during the previous stage of enzymatic saccharification, in which the breakdown of the polysaccharide matrix significantly increased the fraction of available phenolic compounds [36]. Subsequently, lactic fermentation mainly acted by preserving or moderately modifying the flavonoids already released, resulting in high values in both types of BF, although without sufficient variations to generate statistical differences. This increase in flavonoids after hydrolysis can be interpreted as the release of flavonoids bound to the cell wall or the conversion of phenolic derivatives to compounds that react with the flavonoid reagent (aluminum), which has been documented in studies where enzymatic hydrolysis increases the fraction of detectable phenolic compounds and flavonoids [77].

The antioxidant capacity was evaluated using the DPPH, ABTS, and FRAP assays, and the results are presented in Table 4. DPPH activity was higher in the fresh sample (99.58 ± 1.72 TE/L) and decreased after drying; hydrolysis and fermentation gave variable results but were generally lower than those of the fresh sample. ABTS activity showed an intermediate pattern in the fresh sample (40.21 ± 0.21 TE/L) and was reduced by drying.

The FRAP assay showed the highest reducing power in the fresh samples (412.92 ± 1.00 TE/L), followed by a drop with drying and hydrolysis, and partial recovery after fermentation. According to the results of DPPH, ABTS, and FRAP, the most effective antioxidant fraction, both in terms of capacity and reducing power, is concentrated in fresh biomass, and part of this capacity is lost through drying processes. Partial recovery through hydrolysis/fermentation suggests the release of compounds with antioxidant capacity, although not necessarily equivalent in mechanism to those present in the untreated sample. The influence of processing on the antioxidant activity of algae showed compatible patterns between drying and certain extractions degrading phenolic compounds, reducing FRAP and DPPH, while enzymatic and fermentative treatments often increase soluble fractions with antioxidant activity, as measured by some assays.

Furthermore, the fermented Huiro negro samples showed no differences in antioxidant capacity according to the methods evaluated. This could be because TFC contributes similarly to antioxidant activity in both conditions [75,78]. Therefore, these studies represent a promising approach for valorizing algal biomass in the production of fermented beverages or functional extracts. However, to accurately attribute the observed changes to specific compounds, it is recommended to complement these investigations with chromatographic analyses (HPLC–DAD/LC–MS) and to extend stability testing under various drying, hydrolysis, fermentation, and storage conditions [79].

## 4. Conclusions

The combined application of enzymatic hydrolysis and lactic fermentation represents a feasible and efficient valorization strategy for *Lessonia spicata*, enabling its transformation into a functional substrate for fermented beverages. Optimization through response surface methodology identified the EC, T, and HT (60 U/g, 60 °C, and 1.9 h) as the most favorable operational conditions for RSC (2.5 g/100 g), releasing and enhancing *Lactobacillus* spp. growing during refrigerated storage. TPC value in FB (A/E) treatment was the highest, suggesting that saccharification and fermentation act synergistically to improve the bioactive content of the final product.

The composition characterization confirmed that enzymatic hydrolysis enhanced the availability of fermentable carbohydrates, while fermentation contributed to the conversion of macroalgal components into potentially more bioavailable metabolites. The integration of cellulosic saccharification and lactic fermentation constitutes a sustainable and biotechnologically viable pathway for the valorization of *Lessonia spicata*, adding value to this marine resource and supporting the development of novel functional marine-based beverages. Nevertheless, further research should investigate the in vivo bioavailability of bioactive compounds, sensory acceptability, and gut microbiota modulation to validate the potential health benefits and commercial feasibility of the developed product. For instance, simulated gastrointestinal digestion could be employed to better understand the stability and release of bioactive compounds during digestion.

## Figures and Tables

**Table 1:** Experimental runs with coded levels of enzyme concentration (EC, U/g), temperature (T, °C), and hydrolysis time (HT, h).

Runs	Factors		
	EC (U/g)	T (°C)	HT (h)
1	0	0	0
2	0	0	0
3	−1	−1	0
4	1	−1	0
5	−1	1	0
6	1	1	0
7	−1	0	−1
8	1	0	−1
9	0	0	0
10	−1	0	1
11	1	0	1
12	0	−1	−1
13	0	1	−1
14	0	−1	1
15	0	1	1
16	0	0	0
17	0	0	0
18	−1	−1	0
19	1	−1	0
20	−1	1	0
21	1	1	0
22	−1	0	−1
23	1	0	−1
24	0	0	0
25	−1	0	1
26	1	0	1
27	0	−1	−1
28	0	1	−1
29	0	−1	1
30	0	1	1

Runs 1–15: Block 1 and Runs 16–30: Block 2. Each run was performed in triplicate. The experimental design included three factors: enzyme concentration (EC, 10-35-60), temperature (T, 40-50-60), and hydrolysis time (HT, 15-2.5-3.5)—each evaluated at three coded levels (−1, 0, +1), including replicated center points.

**Table 2:** Effect of EC, T, and HT on the release of reducing sugar content (g/100 g).

	Reducing Sugars (g/100 g)	
Factors	Regression Coefficient (β)	*p*-Value
EC	−0.0313	0.0000
EC^2^	0.0007	0.0000
T^2^	0.0011	0.0485
HT^2^	−0.1483	0.0109
EC∙T	0.0005	0.0310
EC∙HT	−0.0048	0.0238
R^2^	96.8807	
R^2^_Adjusted_	95.1477	
MAPE	0.0857	
DW	2.2098	
MAPE = Mean Absolute Percentage Error. DW = Delta error.		

**Table 3:** Nutritional properties (wet basis) at different stages and conditions of Huiro negro treatments.

Composition (g/100 g Wet Basis)	Huiro Negro		
	Hydrolyzed	FB (A/E)	FB (W/E)
Moisture	96.50 ± 0.52 ^b^	97.30 ± 0.05 ^a^	97.20 ± 0.01 ^a^
Fat	0.25 ± 0.04 ^b^	0.48± 0.02 ^a^	0.45 ± 0.05 ^a^
Protein	0.61 ± 0.11 ^a^	0.40± 0.01 ^a^	0.39 ± 0.02 ^a^
Ash	0.29 ± 0.25 ^a^	0.31± 0.03 ^a^	0.33 ± 0.01 ^a^
Total dietary fiber	0.81 ± 0.03 ^a^	0.85 ± 0.01 ^a^	0.90 ± 0.02 ^a^
Available carbohydrates	1.79 ± 0.01 ^a^	0.64 ± 0.02 ^b^	0.52± 0.03 ^c^

Different lowercase letters (a, b, and c) in the same row indicate significant differences (*p* ≤ 0.05) among Huiro negro treatments.

**Table 4:** Bioactive compounds and AC (wet basis) at different stages and conditions of Huiro negro treatments.

Bioactive Compounds and Antioxidant Capacity	Huiro Negro				
	Untreated	Dried	Hydrolyzed	FB (W/E)	FB (A/E)
TPC (mg GAE/L)	156.23 ± 3.04 ^a^	99.46 ± 0.63 ^d^	113.00 ± 1.05 ^c^	118.05 ± 2.94 ^c^	126.59 ± 5.58 ^b^
TFC (mg QE/L)	37.82 ± 0.18 ^c^	61.52 ± 1.00 ^b^	111.41 ± 0.73 ^a^	112.10 ± 0.90 ^a^	113.53 ± 3.18 ^a^
DPPH (μmol TE/L)	99.58 ± 1.72 ^a^	84.80 ± 2.35 ^b^	84.03 ± 1.62 ^b^	86.60 ± 0.56 ^b^	86.79 ± 3.34 ^b^
ABTS (μmol TE/L)	40.21 ± 0.21 ^a^	32.36 ± 0.59 ^b^	40.12 ± 0.13 ^a^	39.94 ± 0.16 ^a^	40.05 ± 0.07 ^a^
FRAP (μmol TE/L)	412.92 ± 1.00 ^a^	384.84 ± 3.89 ^b^	369.73 ± 8.72 ^c^	384.77 ± 0.68 ^b^	390.77 ± 3.03 ^b^

A/E: Added enzyme; W/E: Without enzyme; TPC: Total phenolic content; TFC: Total flavonoid content. Different lowercase letters (a, b, c, d) in the same row indicate significant differences (*p* ≤ 0.05) among Huiro negro treatments.

## Data Availability

The authors declare that the data supporting this study are associated with internal institutional projects DI N° 039.486/2024 and N° 656039.722/2025. These projects are subject to ongoing development and institutional data-protection policies; therefore, access to the datasets is currently restricted.
